# What is the relationship between the coronavirus crisis and air pollution in Tunisia?

**DOI:** 10.1007/s41207-020-00189-5

**Published:** 2020-11-04

**Authors:** Nihel Chekir, Yassine Ben Salem

**Affiliations:** National Engineering School of Gabes, University of Gabes, Gabes, Tunisia

**Keywords:** COVID-19, Tunisia, Air pollution, Lockdown, Environment, Gas emissions

## Abstract

**Abstract:**

Since the beginning of 2020, the COVID-19 pandemic has generated horror and panic around the world. Nevertheless, this terrible crisis is having a positive side effect: it is lowering pollution levels. The outbreak of the coronavirus has caused many governments to impose measures to slow the spread of the virus within populations, such as limiting population displacement, requesting social distancing and the isolation of individuals at home, and reducing industrial activity. In this work, we investigated the effects of governmental measures taken to limit the spread of COVID-19 on the concentrations of air pollutants over four Tunisian cities (Tunis, Sousse, Sfax, and Tataouine). Data on the average daily levels of nitrogen dioxide, sulfur dioxide, carbon monoxide, and particulate matter during January, February, March, and April of 2020 were collected, treated, and analyzed for each city. Curves of average monthly pollutant concentrations from 1 January to 30 April for each city investigated showed that measures taken to reduce the spread of the virus had a substantial impact on emission levels: there were tremendous drops of 51% in NO_2_ and 52% in SO_2_ over Sfax City during March compared to those during January, while nitrogen dioxide and sulfur dioxide levels dropped by about 38% and 42%, respectively, over Tunis City and by around 20% for Sousse. During the four months investigated, almost all of the pollutant concentrations showed a significant drop from mid-March. On 12 March, the Tunisian government imposed some individual and collective measures to protect the population from the virus, such as social distancing, limiting transportation, shutting down schools and universities, and reducing industrial activity. A general lockdown was brought in later. Thus, restricting human and industrial activities appeared to affect the air quality in Tunisia, leading to a marked improvement in the air quality index.

**Graphic abstract:**

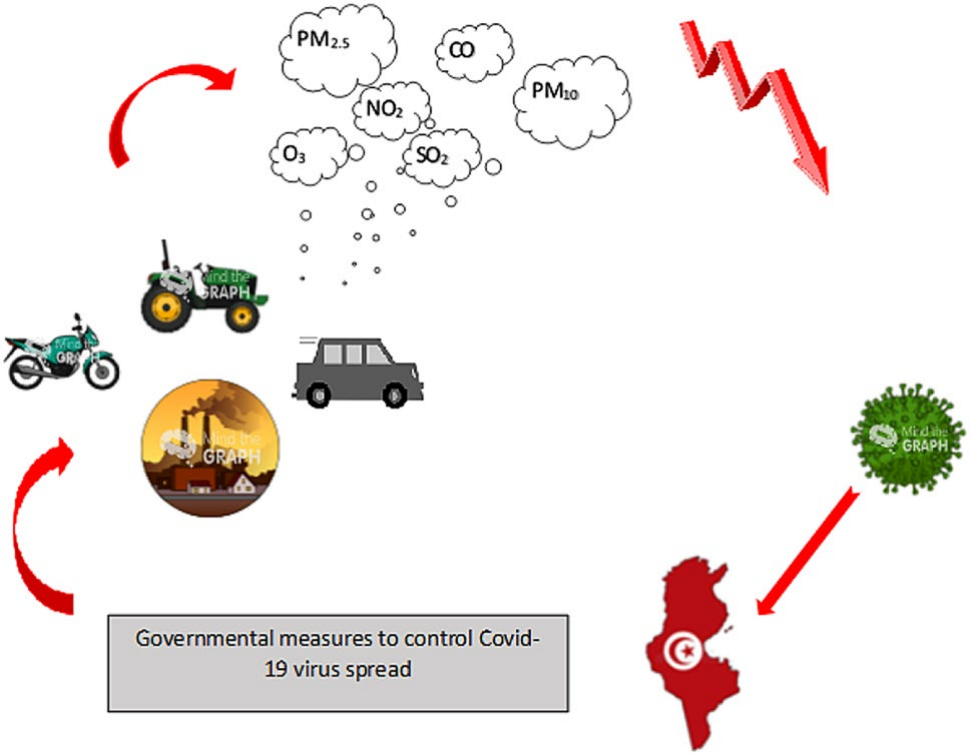

## Introduction

As of 30 April 2020, the novel coronavirus SARS-CoV-2, which originated in China in late December 2019, was reported to have infected 3,266,132 people around the world and killed almost 234,000 since the start of the COVID-19 (COronaVIrus Disease 2019) pandemic (Worldmeters 2020). This pandemic has affected almost all countries, although it is most prevalent in America, followed by Europe, Asia, and the Middle East. For instance, by 30 April 2020, there had been at least 994 confirmed cases of the novel coronavirus in Tunisia, including 41 deaths (Worldmeters 2020).

The World Health Organization (WHO) has warned countries that the COVID-19 pandemic requires urgent action. Moreover, they have been preparing for the worst-case scenario as the total number of virus infections has increased. This scenario, as reported by a former chair of the Global Health Council and a long-term collaborator with the World Health Organization (WHO) (Spinney 2020), is that the outbreak goes global and the disease eventually becomes endemic, meaning that it circulates permanently in the human population.

Due to the recent rapid emergence of the global COVID-19 pandemic, the World Health Organization has proposed emergency measures that aim to reduce virus transmission rates and contamination. These measures consist of limiting population movement and travel, strict social distancing, the isolation of individuals at home, and even, if necessary, a general population lockdown (Lai 2020). While this suppression of human activity has many negative effects, it is a critically important way of limiting the spread and impact of COVID-19 (Coccia 2020). On the other hand, the marked reduction in human activity caused by these measures appears to have coincided with an improvement in the air quality in cities, so this substantial change in human behavior is thought to have altered air pollution levels (He 2020; Cadotte 2020).

During the second half of March 2020, gas emissions over China dropped by a quarter as cars, trucks, power plants, and factories ground to a halt. The same phenomenon was seen in California (which is known to have very high levels of air pollution), as shown by images taken by NASA’s Earth-observing satellite. Scientists predict that global carbon dioxide emissions could drop by more than 5% in 2020, which would represent the first decrease in carbon dioxide emissions since the 2008 economic crisis (when it fell by 1.4%), and the most significant fall during the past 50 years (Reuters 2020). Satellite images have highlighted noticeably decreased levels of nitrogen dioxide in large industrial cities and regions in Europe (e.g., Brussels, Paris, Madrid, Milan, Frankfurt, and northern Italy) during the period 5–25 March 2020 as compared to the same period in 2019 (Wood 2020).

Industrialization and increasing numbers of vehicles are worsening levels of air pollution around the world. In fact, the air has become so polluted that it can no longer be cleaned up by natural processes (IQAir 2020). The Environmental Protection Agency (EPA) has defined the six main contributors to air pollution (EPA 2014): ground-level ozone (O_3_), particle pollution, lead, nitrogen dioxide (NO_2_), carbon monoxide (CO), and sulfur dioxide (SO_2_). These air pollutants, which are considered to be critical emissions, are found in urban areas and released into the atmosphere at concentrations high enough to gradually induce severe health problems (EPA 2010).

Air pollution affects human health in many different ways (Sanità 2020). Exposure to high levels of air pollutants for a short period can produce short-term health effects that mainly affect the eyes, throat, nose, and upper respiratory system, causing asthma, emphysema, pneumonia, and bronchitis. Other symptoms include nausea, headache, and allergies. On the other hand, exposure to low levels of air pollutants over a long period can lead to long-term effects such as heart disease, lung cancer, chronic respiratory disease, and even damage to the brain, liver, nerves, or kidneys.

Recent scientific studies conducted in some countries and cities have examined the effects of the COVID pandemic on air pollution (Anjum 2020). Improvements in air quality were observed in China (He 2020), Italy (Lippi 2020), England (Travaglio 2020), Brazil (Nakada 2020; Dantas 2020), Kazakhstan (Kerimray 2020), India (Sharma 2020), and worldwide (Shrestha 2020).

In the present study, we investigated how the levels of six air pollutants in four Tunisian cities varied during January, February, March, and April 2020 to probe the effects of the COVID-19 pandemic crisis and protection measures imposed by the Tunisian government, including the lockdown. These cities have different characteristics in terms of their urban/rural nature, traffic levels, and residential/industrial character. We collected, treated, and analyzed freely available air monitoring data for the selected cities to assess the impact of the coronavirus outbreak on urban air pollution.

## Data and method

### Investigated cities

**Fig. 1:**
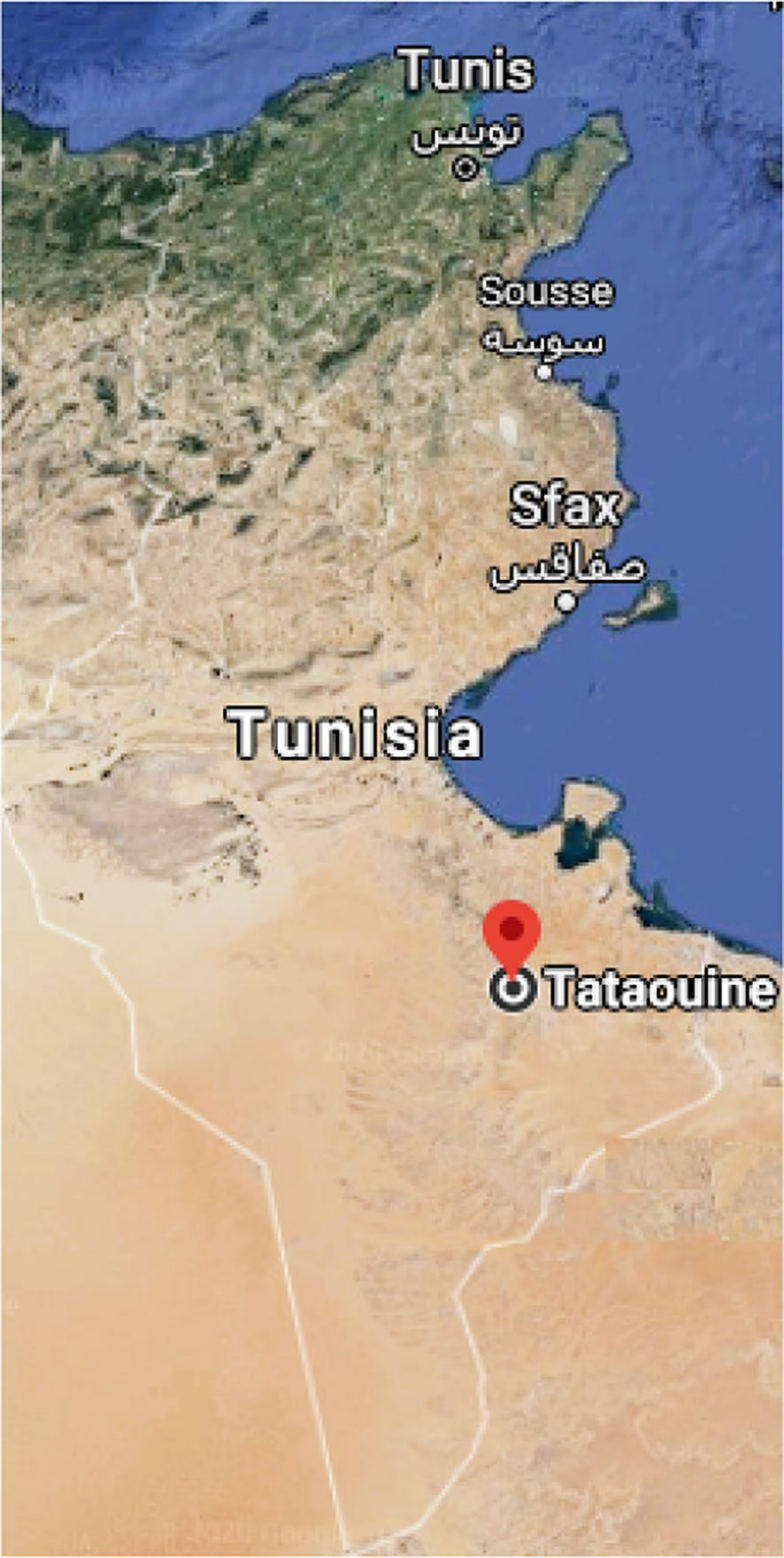
Locations of the four Tunisian cities investigated in this work

Tunisia is a country in the Maghreb region of North Africa that covers 163,610 square kilometers and had a population of 11.582 million in 2018 (World Bank 2020). Four Tunisian cities that were affected by COVID-19 early during the pandemic were selected for this study. The four cities were chosen based mainly on location, economic activity, and population. The cities were the capital Tunis in the north of Tunisia, Sousse and Sfax, both of which are in the center of the country, and Tataouine, which is in southern Tunisia. The map shown in Fig. 1 indicates the locations of the investigated cities.

The four studied cities have different characteristics. Tunis, Sousse, and Sfax are highly populated urban regions with considerable industrial activity (Belhedi 2007). According to the 2014 population census, Tunis (the capital of Tunisia) has the most inhabitants (1,056,247) of any Tunisian city, followed by Sfax and Sousse, which are ranked the second and the fourth largest cities in Tunisia in terms of population, with 955,421 and 674,971 inhabitants, respectively (National Statistics Institute 2014). Another factor taken into consideration in this study was the date of the first confirmed COVID-19 case in the city. The selected cities were infected early during the pandemic (during the period 9–18 March) (Tunisian Ministry of Health 2020). For each city, we collected air pollution data and analyzed pollutant trends before and after the actions taken by the government on 12 March 2020 to contain the spread of the virus. A four-month period was examined: January, February, March, and April 2020. Table 1 lists relevant characteristics of the investigated cities and the dates of the first confirmed infections in those cities.

**Table 1:** Characteristics of the investigated cities (Villeret 2020)

City	Urban or rural?	Population in 2014	Amount of vehicular traffic	Main economic sector(s)	Date of the first confirmed case
Tunis	Urban	1,056,247	High	Industrial/power plant	9 March 2020
Sousse	Urban	674,971	High	Industrial/power plant/agricultural	16 March 2020
Sfax	Urban	955,421	High	Industrial	18 March 2020
Tataouine	Urban/rural	149,453	Low	Agricultural	14 March 2020

The main sources of industrial activity in Tunis are ICT, the ecoindustry, aeronautics, and pharmaceuticals. There is also a power plant in Rades, a suburban region of Tunis. Sousse, which is characterized by its mechanical and electronic industries, also has a power plant. Sfax, which is the second most important city in Tunisia from an economic perspective, is known for its chemical industry. The two power plants in Rades and Sousse are owned by the Tunisian Electricity and Gas Company (STEG), a public company that is charged with the generation and distribution of electricity. As reported in the 2018 STEG Annual Report, electricity is mostly produced by fossil fuel-powered units (94%) in Tunisia.

### Air pollution data collection

In this study, we collected air quality index (AQI) data and air pollutant concentrations from the Air Matters monitoring platform, a global air quality service provider (www.air-matters.com) that forecasts and provides real-time information on the air quality index, allergenic pollen levels, and weather data. For each investigated city, we downloaded the average daily AQI values and concentrations of the six major air pollutants for the four-month period from 1 January to 30 April 2020. The pollutants considered were fine particulate matter (PM_2.5_), coarse particulate matter (PM_10_), nitrogen dioxide (NO_2_), ground-level ozone (O_3_), sulfur dioxide (SO_2_), and carbon monoxide (CO).

**Fig. 2:**
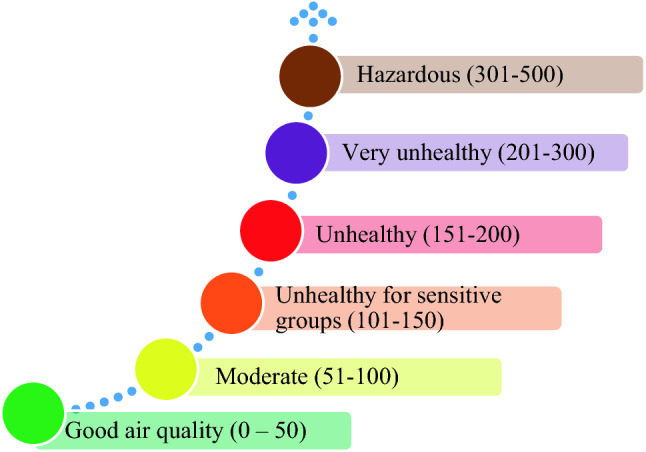
Air quality index classification according to the EPA

The air quality index (AQI) relates daily pollutant concentrations to health concerns for sensitive groups and the public (EPA 2014). Values below 100 are considered satisfactory. Values above 100 are considered unhealthy—initially for certain sensitive groups of people, and then for everyone as the AQI values increase further. The AQI can range from 0 to 500, and this range is divided into six classes, as shown in Fig. 2.

### Data analysis

For each investigated city, we used the data collected daily to determine the average monthly air quality index and the average monthly concentrations of the six air pollutants for January, February, March, and April 2020.

We assumed that the air quality in January and February 2020 (before the pandemic hit Tunisia) was normal. Therefore, changes in air quality during March and April 2020 were considered to be caused primarily by the actions of the country aimed at containing the spread of COVID-19. We examined Tunisian air pollution for the four first months of 2020 and assessed whether there were linear trends in AQI and pollutant concentration values because of the protection and lockdown measures imposed by the government due to the COVID-19 pandemic. We then compared pre- and post-COVID-19 air quality for the affected cities.

## Results

**Fig. 3:**
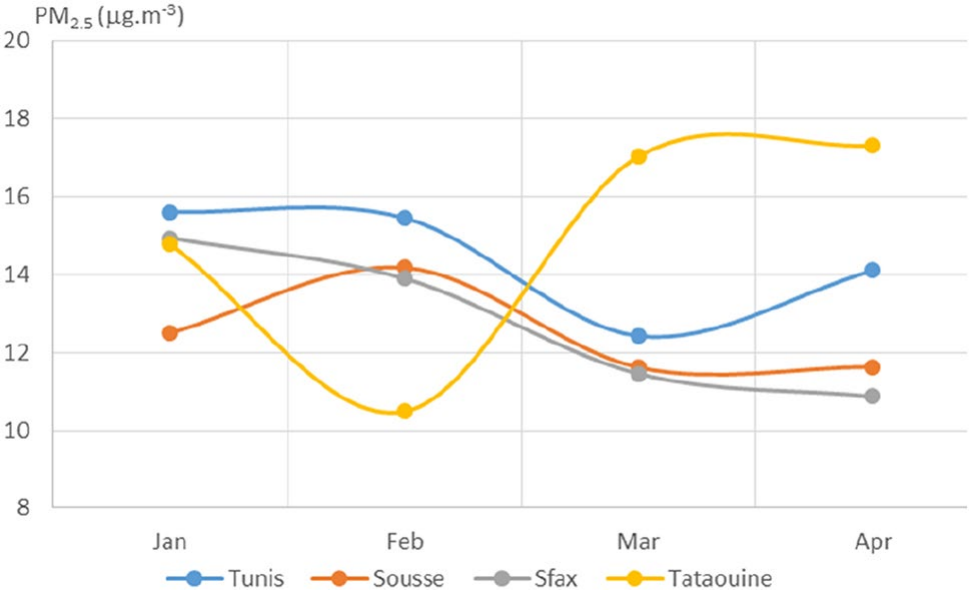
Monthly average concentrations (in μg/m^3^) of PM_2.5_

**Fig. 4:**
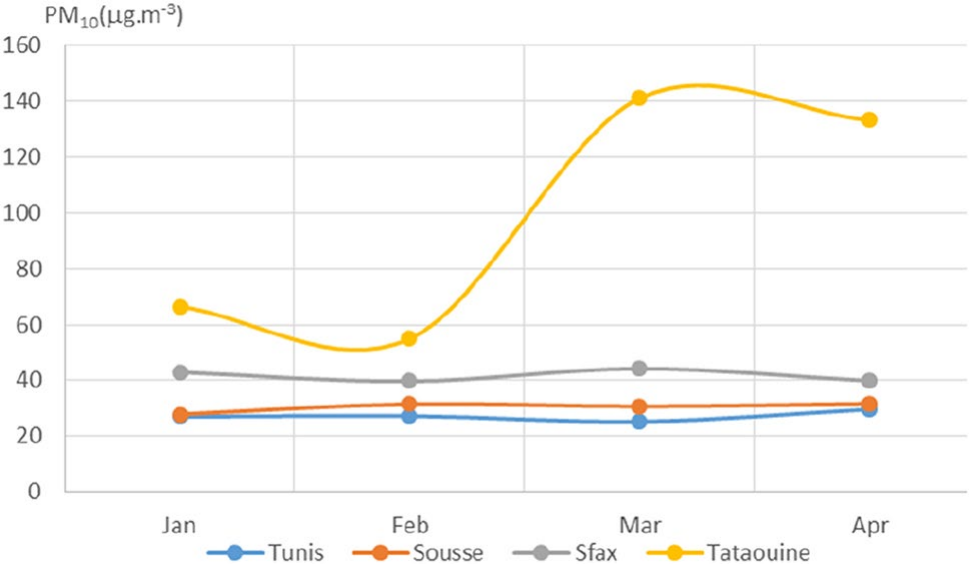
Monthly average concentrations (in μg/m^3^) of PM_10_

**Fig. 5:**
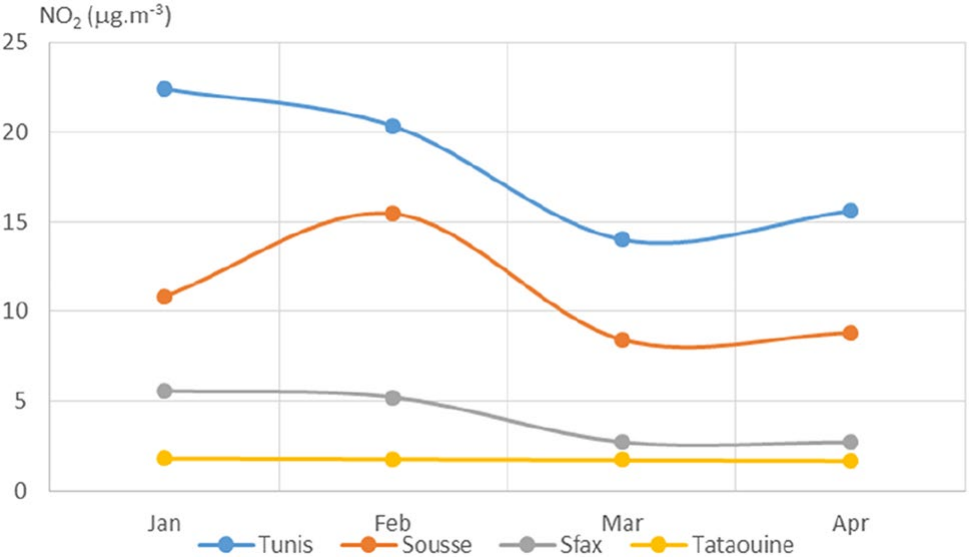
Monthly average concentrations (in μg/m^3^) of NO_2_

**Fig. 6:**
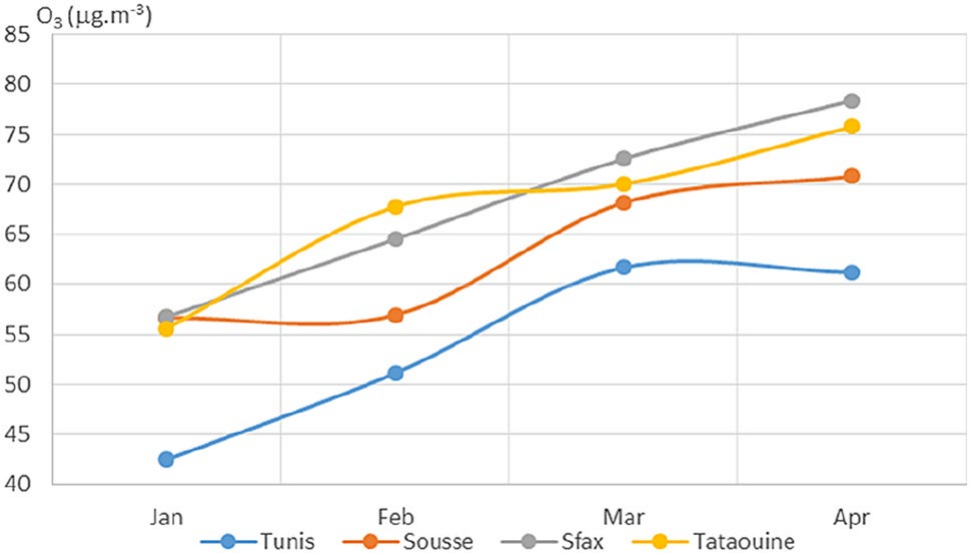
Monthly average concentrations (in μg/m^3^) of O_3_

**Fig. 7:**
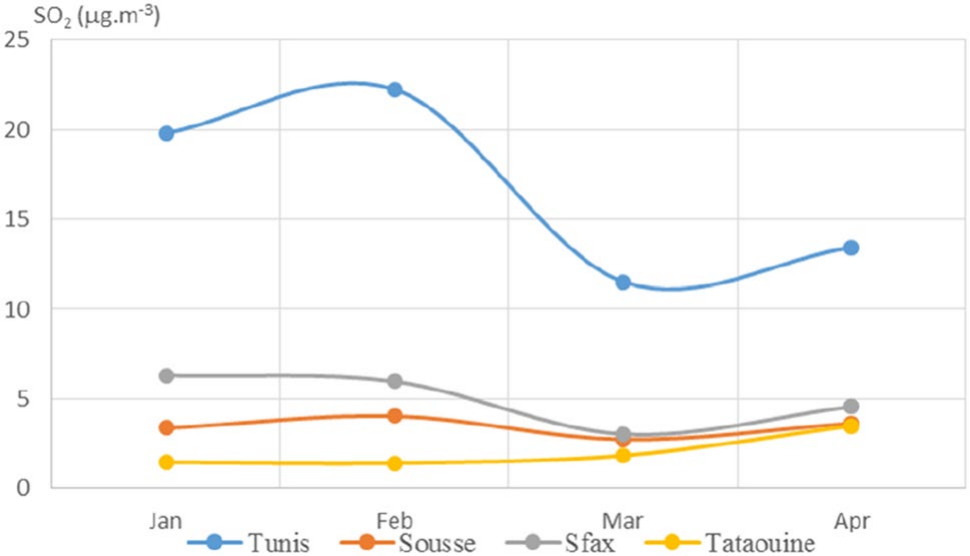
Monthly average concentrations (in μg/m^3^) of SO_2_

**Fig. 8:**
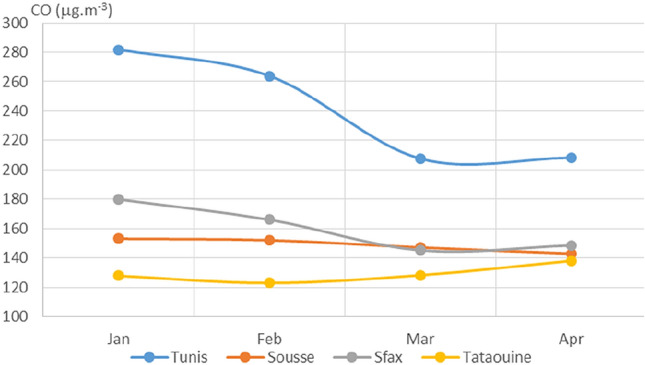
Monthly average concentrations (in μg/m^3^) of CO

**Fig. 9:**
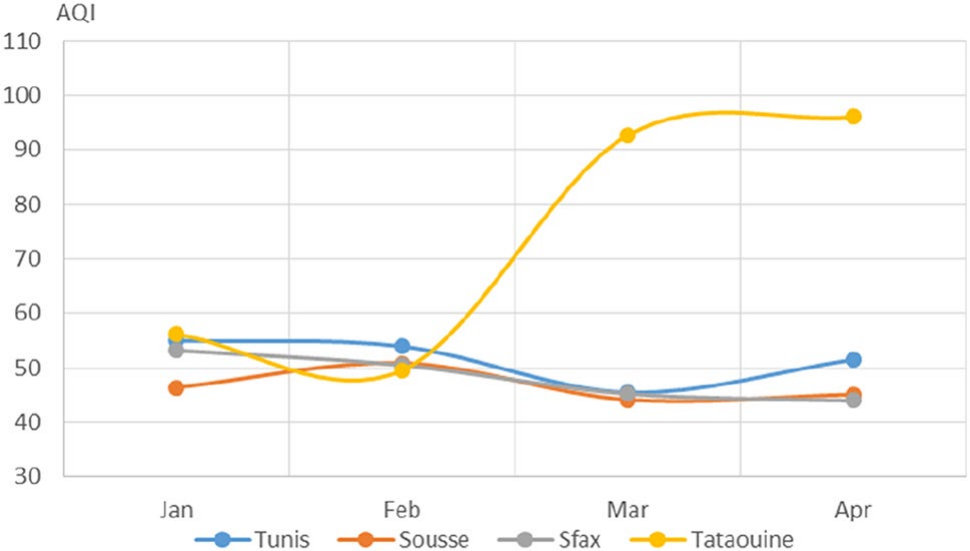
Monthly average AQI values

Plotted average monthly values of PM_2.5_, PM_10_, NO_2_, O_3_, SO_2_, CO, and AQI for the four chosen Tunisian cities are displayed in Figs. 3, 4, 5, 6, 7, 8, and 9, respectively. We compared the levels of the different air pollutants in January and February to those in March and April, when the COVID-19 protection measures were imposed or in place.

The mean concentrations of almost all the pollutants showed significant reductions in every city following the enactment of COVID-19 protection measures: the curves shown in the figures present substantial differences in pollutant levels between the period before early March 2020 and the period after it.

When the concentrations of PM_2.5_ during the four months of interest were analyzed (see Fig. 3), we found that there was a slight drop in March compared to January for all of the cities considered except for Tataouine. The observed drops in March as compared to January for Tunis, Sousse, and Sfax were about 20%, 7%, and 23%, respectively.

No significant change in PM_10_ concentration was observed for Tunis, Sousse, and Sfax, while a significant increase was noted for Tataouine (see Fig. 4).

For NO_2_, the concentration curves for the four cities (see Fig. 5) exhibited decreases in March and April as compared to January and February. Nitrogen dioxide concentrations in March were 37%, 22%, and 51% lower than those in January for Tunis, Sousse, and Sfax, respectively, whereas the NO_2_ concentration over Tataouine remained constant across the period of interest. As depicted in Figs. 7 and 8, very similar behavior was observed for the levels of SO_2_ and CO, as decreased concentrations were observed shortly after the restrictions for almost all of the cities. The measured concentration of SO_2_ in Tunis, Sousse, and Sfax declined by 42%, 18%, and 52%, respectively, in March as compared to those in January. The levels of carbon monoxide declined by around 26%, 4%, and 19% for Tunis, Sousse, and Sfax, respectively.

The protection measures enacted by the government appeared to cause a drop in air pollution, as indicated by the air quality index (see Fig. 9). The AQI dropped by 17%, 5%, and 15% in March as compared to January and by 6%, 3%, and 17% in April as compared to January for Tunis, Sousse, and Sfax. When compared to the previously mentioned pollutants, ozone showed a late response to the protective measures against COVID-19, which is why the curves shown in Fig. 6 do not follow the same trend as seen for the other pollutants.

## Discussion

From mid-March to the end of April, levels of the pollutants PM_2.5_, NO_2_, SO_2_, and CO as well as AQI values were observed to be substantially lower than those in early January, when power plants and industrial factories were operating at normal levels. The appreciable drop in PM_2.5_ concentrations can be attributed to the reduction in emissions from vehicular traffic and industrial activity caused by the measures brought in by the Tunisian government to suppress the spread of COVID-19. The increase in PM_2.5_ concentrations in Tataouine following the introduction of these measures is thought to be due to the meteorological conditions during this time. Tataouine is a pre-Saharan city—a doorway to the desert in southern Tunisia. Storms from the Sahara during the spring months bring a lot of dust, which explains the increased PM_2.5_ levels observed for March and April.

The remarkable increase in PM_10_ for Tataouine during March and April (see Fig. 4) can also be explained by the weather conditions. In Tunis, Sousse, and Sfax, large particulates are generally produced through human activity. The relatively constant levels of PM_10_ in these cities during the monitored period may be due to the long residence time of PM_10_ in the atmosphere.

For NO_2_, SO_2_, and CO, decreased levels were seen for March and April than for January and February. This appears to have been due to the reduced activities of power plants and other industrial facilities and the restrictions on vehicular use that were imposed from March on to limit the spread of COVID-19. This impact of restrictions due to COVID-19 on pollutant concentrations has been noted in various studies performed in cities around the world (Otmani 2020; Shi 2020; Zhao 2020).

Urbanization and motorization are considered two important sources of congestion. They consume energy and therefore generate pollutant emissions. This explains why the capital Tunis exhibits the highest mean values of the pollutants SO_2_, NO_2_, CO, and PM_2.5_.

The only city that showed nonsignificant changes in NO_2_, SO_2_, and CO concentrations following the introduction of the measures aimed at tackling COVID-19 was Tataouine. The increased concentrations of O_3_ in all of the cities in March and April can be attributable to the meteorological conditions, because O_3_ levels tend to rise during spring and summer owing to increased solar radiation (in terms of its intensity and daily duration) (Escudero et al. 2019; Wang et al. 2020). Similar behavior of ozone was observed in Milan (Collivignarelli 2020).

**Fig. 10:**
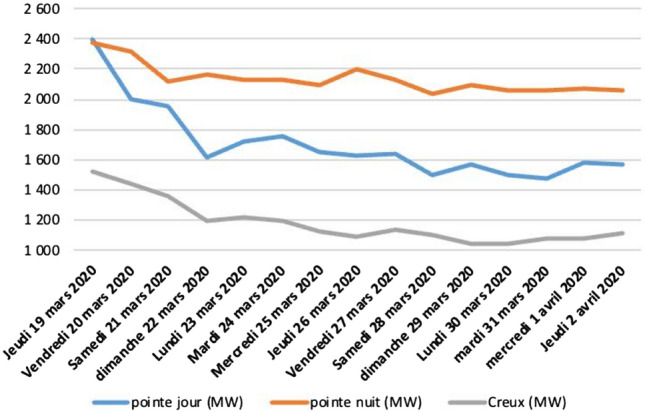
Electricity demand (MW)

**Fig. 11:**
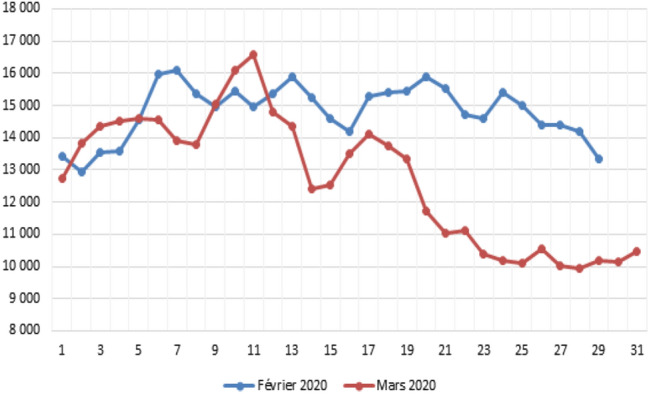
Daily demand for natural gas (TEP)

The general air quality improved in March and April, as inferred from the trends in the air quality index (Fig. 9). This general shift in air pollutant concentrations would be expected given that the governmental measures relating to the COVID-19 pandemic were introduced on 12 March. The resulting partial lockdown of the Tunisian population and partial shutdown of industrial activity changed individual and collective behavior. The number of vehicles used dropped dramatically. Moreover, electricity production until 4 April was about 25% lower than that for the same period in 2019. As can be seen in Figs. 10 and 11 in Appendix A, the demand for electricity and natural gas was also significantly reduced during lockdown and shutdown (Ministère EMTE 2020).

Due to the pandemic, the Tunisian authorities progressively shut down transportation and travel in and out of the country. They also curtailed local business travel, limited local transport, and closed down schools, colleges, and universities to reduce the spread of the disease, as well as establishing numerous quarantine sites in infected areas. Those governmental measures taken in March (limiting transport and shutting down schools on 12 March) rapidly and markedly reduced human movement and activity. The social distancing and almost complete cessation of economic activity caused by the COVID-19 measures led to a significant drop in air pollution over the four investigated towns.

When analyzing the effects of the protection measures and lockdown on the air pollution in the studied cities, we observed that the impact varied depending on the level of industrial activity usually associated with the city.Tataouine, which has an economy that is largely based on agriculture, showed notably different trends in pollutant levels than the other more industrial cities. The concentrations of pollutants in the industrialized cities were more strongly influenced by the COVID-19 measures. Similarly, the air pollution in big cities that usually consume more electricity or suffer from substantial traffic was particularly strongly reduced when the lockdown was implemented.

## Conclusion

Since 12 March 2020, the Tunisian government has taken a series of protection measures to limit the spread of the novel coronavirus among the Tunisian population. These unique circumstances are perfect for studying the impact of human behavior on air pollution in Tunisia. We carried out the present study to investigate the possible impacts of the COVID-19 human lockdown in Tunisia on air pollution. We utilized average daily air pollutant concentrations collected during air quality monitoring for four cities in Tunisia (Tunis, Sousse, Sfax, and Tataouine). Analysis of the trends in monthly average air pollutant concentrations during the first four months of 2020 showed major drops in PM_2.5_, NO_2_, SO_2_, and CO levels from 12 March, when the protection measures were first imposed. A comparison of air quality status between the four Tunisian cities before and after this date revealed that the concentrations of almost all of the pollutants dropped after the measures were introduced. The concentration of PM_2.5_ reduced over each city except for Tataouine. The concentration of larger particulates (PM_10_) did not change significantly in Tunis, Sousse, and Sfax over the period of interest. However, for Tataouine, the southernmost city, PM_10_ increased during March and April because of the prevailing meteorological conditions. We also noted marked decreases in the concentrations of nitrogen dioxide, sulfur dioxide, and carbon monoxide in almost all the cities. The largest reduction was observed for the capital Tunis. On the other hand, notable increases in O_3_ were seen in all the studied cities. The general air quality as assessed using the air quality index appeared to improve in almost all the cities following the introduction of the COVID-19 measures.

Thus, our study shows that the progressive limitation of industrial activity and vehicular traffic and the drop in electric power demand in Tunisia caused by the measures brought in to address the COVID-19 pandemic were accompanied by apparent decreases in air pollution and the air quality index in Tunisian cities. In other words, one side effect of this horrific virus is cleaner Tunisian air.

## Appendix A

See Figs. 10 and 11.

## Abbreviations

AQI: Air quality index
CO: Carbon monoxide
COVID: Coronavirus disease
EPA: Environmental Protection Agency
ICT: Information and communication technology
MERS: Middle East respiratory syndrome
NASA: National Aeronautics and Space Administration
NO_2_: Nitrogen dioxide
PM: Particulate matter
SARS: Severe acute respiratory syndrome
SO_2_: Sulfur dioxide
STEG: Tunisian Electricity and Gas Company
VOC: Volatile organic compounds
WHO: World Health Organization
